# Opposite Effects of the S4–S5 Linker and PIP_2_ on Voltage-Gated Channel Function: KCNQ1/KCNE1 and Other Channels

**DOI:** 10.3389/fphar.2012.00125

**Published:** 2012-07-05

**Authors:** Frank S. Choveau, Fayal Abderemane-Ali, Fabien C. Coyan, Zeineb Es-Salah-Lamoureux, Isabelle Baró, Gildas Loussouarn

**Affiliations:** ^1^UMR 1087, Institut National de la Santé et de la Recherche MédicaleNantes, France; ^2^UMR 6291, Centre National de la Recherche ScientifiqueNantes, France; ^3^L’Institut du Thorax, L’UNAM Université, Université de NantesNantes, France

**Keywords:** voltage-gated potassium channels, S4–S5 linker, phosphatidylinositol 4,5-bisphosphate, patch-clamp, channelopathies

## Abstract

Voltage-gated potassium (Kv) channels are tetramers, each subunit presenting six transmembrane segments (S1–S6), with each S1–S4 segments forming a voltage-sensing domain (VSD) and the four S5–S6 forming both the conduction pathway and its gate. S4 segments control the opening of the intracellular activation gate in response to changes in membrane potential. Crystal structures of several voltage-gated ion channels in combination with biophysical and mutagenesis studies highlighted the critical role of the S4–S5 linker (S4S5_L_) and of the S6 C-terminal part (S6_T_) in the coupling between the VSD and the activation gate. Several mechanisms have been proposed to describe the coupling at a molecular scale. This review summarizes the mechanisms suggested for various voltage-gated ion channels, including a mechanism that we described for KCNQ1, in which S4S5_L_ is acting like a ligand binding to S6_T_ to stabilize the channel in a closed state. As discussed in this review, this mechanism may explain the reverse response to depolarization in HCN-like channels. As opposed to S4S5_L_, the phosphoinositide, phosphatidylinositol 4,5-bisphosphate (PIP_2_), stabilizes KCNQ1 channel in an open state. Many other ion channels (not only voltage-gated) require PIP_2_ to function properly, confirming its crucial importance as an ion channel cofactor. This is highlighted in cases in which an altered regulation of ion channels by PIP_2_ leads to channelopathies, as observed for KCNQ1. This review summarizes the state of the art on the two regulatory mechanisms that are critical for KCNQ1 and other voltage-gated channels function (PIP_2_ and S4S5_L_), and assesses their potential physiological and pathophysiological roles.

## Part 1: Role of the S4–S5 Linker in Channel Voltage Dependency

Voltage-gated ion channels are all designed according to a common pattern including six transmembrane segments (S1–S6), with S1–S4 forming the voltage-sensing domain (VSD), in which the positively charged S4 is the voltage sensor *per se*, and S5–S6 forming the pore. The N- and C-termini are cytosolic. Whereas voltage-gated K^+^ channels are tetrameric assemblies of identical or homologous subunits, eukaryotic Ca^2+^ and Na^+^ voltage-gated channels are the result of the fusion of four subunits.

If a general consensus started to emerge on the nature and the (nano-)metrics of the movement of the voltage sensor in voltage-gated channels, it is still not the case for the nature of the coupling between the voltage sensor movement and the gate opening. In this first part, we review most of the results obtained through various experimental approaches on various channels, that can give insights on the nature of the coupling, and we try to classify this coupling into two categories: a strong or a labile coupling between the main actors, namely, the S4–S5 linker (referred here as S4S5_L_) and the C-terminal part of the S6 transmembrane segment (S6_T_).

### How does the voltage sensor regulate pore gating?

#### Movement of the voltage sensor

The ability of K_v_ channels to sense the membrane potential is conferred via the VSD. The S4 segment moves across the plasma membrane in response to changes in membrane potential, allowing the transition of the channel between a closed conformation and an open conformation. Many studies have investigated the nature of the S4 movement, and came up with three different models, with major differences in this movement.

* According to the crystal structure of KvAP, S4 coupled to S3 form a helical hairpin, or “paddle,” moving 15–20 Å across the lipid bilayer, as confirmed by avidin accessibility to different-length tethered biotin reagents (Jiang et al., 2003; Ruta et al., 2005). However, a number of lines of evidence suggest that the KvAP structure does not correspond to a native conformation, such as the fact that the VSD is in a resting state and in contrast, the pore is in the open state. This non-native state is potentially due to the use of monoclonal antibody fragments in order to stabilize the structure, but more likely due to the absence of membrane lipids (Lee et al., 2005).
* The “transporter” model, described in Shaker, involves a very small movement of S4 (2–3 Å) from a crevice in contact with the intracellular solution to another one in contact with the extracellular solution (Cha et al., 1999; Chanda et al., 2005).
* Finally, the helical screw model (Guy and Seetharamulu, 1986) and the similar sliding helix model (Catterall, 1986), originally proposed for sodium channels, have been then adapted to K_v_ channels (Durell and Guy, 1992). These models suggest that S4 rotates ~180°, and at the same time, translates ~13.5 Å along its axis (reviewed in Börjesson and Elinder, 2008). Strikingly, “embryonic” paddle and helical screw models were predicted as early as 1981 (Figures 2 and 9 of Armstrong, 1981).

The variety of these models, and the fact that they predict a magnitude of the S4 movement across the membrane ranging from 2 Å (Cha et al., 1999) to ~15 Å in Shaker (Larsson et al., 1996), most probably come from the variety of the techniques employed. Some of the techniques (such as FRET) underestimate the distances by capturing rare conformations when the donor and acceptor are nearby. On the contrary, cross-linking or tethered biotin may overestimate distances by capturing and covalently stabilizing rare and extreme conformations in which a cysteine is accessible intracellularly or extracellularly (Tombola et al., 2006).

A structural model of Shaker, based on the crystal structure of Kv1.2, predicts an axial rotation and a translation of S4 (Yarov-Yarovoy et al., 2006) as described in the “helical screw” model. In addition, a subsequent tilting motion of the S4 is also suggested. Very recently, disulfide-locking experiments and structural models of resting and activated state of the VSD in a sodium channel, NaChBac, propose an outward movement (~6–8 Å) of S4 relative to S1, S2, and S3 and a rotation (~30°) of the S4 coupled to a tilting motion relative to the S4S5_L_ (Yarov-Yarovoy et al., 2012). Studies of these different channels support the idea that a common S4 movement may be applied to various channels, including KCNQ channels. The next step will be to solve the crystal structure of KCNQ channels, in an attempt to gain insights on the structural conformation corresponding to gating of these channels. Presently, homology models (Smith et al., 2007) and molecular dynamics are valuable templates to better understand the physiological and pathophysiological mechanisms of voltage dependency (Delemotte et al., 2011).

#### Coupling between the voltage sensor and the gate: S4S5_L_ and S6_T_ play a major role

##### The S4S5_L_ interacts with S6_T_ in many voltage-gated channels

Which part of the channel links the voltage sensor movement to the gate opening? The physical interaction between S4S5_L_ and S6_T_ and the role of this interaction in translating the voltage sensor movement to the gate opening have been investigated in many voltage-gated channels by diverse techniques. The results obtained will be detailed below and in other reviews of the present Frontiers Research Topic, but it is important to note that many works stress the major role of this S4S5_L_–S6_T_ interaction. Mutagenesis associated to functional studies using chimeras of Shaker and KcsA (Lu et al., 2001, 2002), alanine-scanning of S4S5_L_ and S6_T_ in Kv4.2 (Barghaan and Bähring, 2009), cross-linking studies of S4S5_L_ and S6_T_ in human ether-a-go-go related gene (hERG; Ferrer et al., 2006), but also in the hyperpolarization-activated channel, spHCN1 (Prole and Yellen, 2006), all converge to the notion that S4S5_L_ and S6_T_ play a major role in the coupling between the voltage sensor and the S6_T_. This is further confirmed by the crystal structures of K_v_ and Na_v_ channels, which show that the distance between S4S5_L_ and S6_T_ fits with the hypothesis that these regions contact each other (Long et al., 2005; Payandeh et al., 2011).

These studies strongly indicate that the VSD-activation and gate coupling are associated through the S4S5_L_–S6_T_ interaction. However, many questions remain to be elucidated. Are other regions of channels involved in this coupling? How exactly does this S4S5_L_–S6_T_ interaction make the link between VSD-activation and gate coupling?

##### The N-terminus and the S1 segment are also involved in the VSD-pore coupling

In addition to S4S5_L_ and S6_T_ interaction, other regions influence voltage-dependent channel activity. One of those regions is the N-terminus (Nter). In many signaling proteins, a PAS domain is present where it functions as a signal sensor and its name comes from the transcription factors in which it was first identified: period circadian protein (Per), aryl hydrocarbon receptor nuclear translocator protein (Arnt), and single-minded protein (Sim). The PAS domain is also present in the N-terminus of three K_v_ channel families, Kv10, Kv11, and Kv12. An interaction between the PAS domain and S4S5_L_ has been postulated as underlying the slow deactivation process of hERG channels (Wang et al., 1998a; Chen et al., 1999). Long QT syndrome (LQT) is a cardiac disease characterized by prolonged ventricular repolarization, arrhythmias, and sudden death. In some LQT patients, a disruption of the presumed interaction between the PAS domain and S4S5_L_ would result in an acceleration of the deactivation rate, leading to a decrease in this critical repolarizing current (Chen et al., 1999). Alonso-Ron et al. (2008) showed that channels lacking Nter domain or bearing mutations in S4S5_L_ exhibited similar slowed deactivation and positive shift in the voltage dependence of activation, supporting the hypothesis of an interaction between these two regions. However, no experiment has unequivocally demonstrated a direct Nter-S4S5_L_ interaction although a recent study has demonstrated a close proximity between Nter and S4S5_L_ in hERG (de la Peña et al., 2011). To highlight such interaction, the authors introduced cysteines in the PAS domain and in the S4S5_L_ and then tested the effects of applying an oxidizing agent, *tert*-butyl hydroperoxide (TbHO_2_), on channels expressing those cysteines. Formation of disulfide bonds, induced by TbHO_2_, between cysteines introduced in Nter and S4S5_L_, dramatically decreases the tail current. This effect is completely reversed by dithiothreitol, a reducing agent. Taken together, these data indicate that Nter can bind to S4S5_L_, stabilizing the channel in the closed state. A more exhaustive review on the role of cytoplasmic domains (CTD) in voltage-gated potassium channels gating is available in another article of this Frontiers Research topic (Barros et al., 2012).

The coupling between the VSD and the pore may also occur through interactions between transmembrane domains (TMD). Cross-linking studies in Shaker channel showed the proximity between the S4 and S5 segments, and suggested that interactions may be involved in the coupling between the VSD and the pore (Broomand et al., 2003; Gandhi et al., 2003). Also, statistical analysis of K_v_ channels sequences and mutagenesis studies suggest that an interface between the S1 domain and the pore helix, both highly conserved in K_v_ channels, is required for this coupling as well (Lee et al., 2009). Indeed, a tryptophan scanning of residues forming the interaction surface between S1 and the pore helix in Shaker has shown that mutations of those residues affect channels function. Finally, formation of a disulfide bond, forcing the S1-pore helix interaction, leads to an alteration of gating.

In summary, a network of interactions, including Nter, S1, S4S5_L_, and S6_T_, seems to be involved in the coupling between VSD and the pore.

### Interaction between S4S5_L_ and S6_T_: Two models

#### Model 1: mechanical lever model (Figure 1A)

##### Shaker

The Shaker gene from *Drosophila melanogaster* was the first potassium channel to be cloned (Tempel et al., 1987), contributing to the identification of a family of homologous channels in vertebrates (the K_v_ superfamily) and to the understanding of the role of K_v_ channels in human diseases. This channel is one of the most extensively studied voltage-activated ion channels and often serves as a model in the study of voltage dependency. In this tetrameric voltage-gated K^+^ channel, the four VSDs are covalently connected to the S5 segments of the pore region by the S4–S5 linkers, as mentioned above. Kinetic models predicted that the gating mechanism of this channel involves several relatively independent movements of the four VSDs between resting and activated states, followed by a concerted opening transition where the S6 gate moves from a closed to an open state (Bezanilla et al., 1994; Hoshi et al., 1994; Stefani et al., 1994; Zagotta et al., 1994a,b). This model was further confirmed by using a triplet of mutations in the S4 that make the final concerted step rate limiting in the activation pathway, thus rendering it more detectable (Ledwell and Aldrich, 1999).

**Figure 1:**
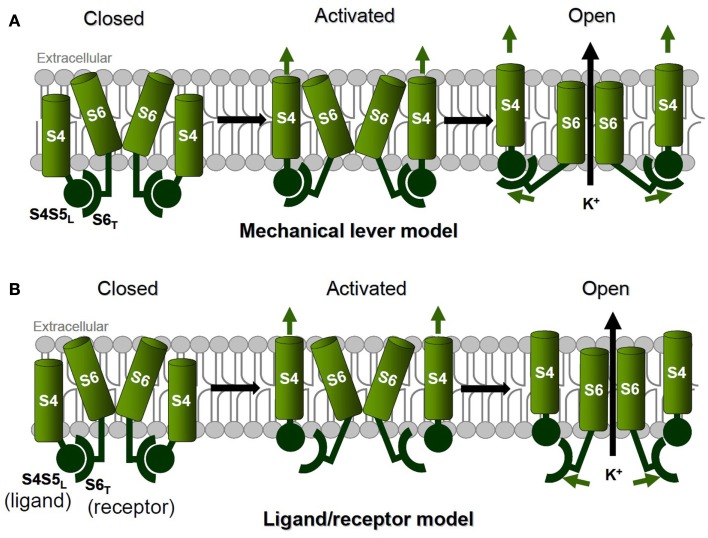
**Two models of coupling between voltage sensor movement and pore opening**. **(A)** The mechanical lever model with a strong coupling between voltage sensor movement and pore opening, as suggested for Kv1.2 and Shaker. **(B)** The ligand/receptor model with a loose and state-dependent coupling between voltage sensor movement and pore opening, as suggested for KCNQ1 channels.

In an elegant work using chimeras in which Shaker pore module is replaced by the one of KcsA channel (Lu et al., 2001, 2002), Lu and co-workers showed that S4S5_L_ and S6_T_ play a key role in voltage dependency. Incomplete channel closures in Shaker-KcsA chimeras with modified S4S5_L_ and S6_T_ suggest that these regions interact in the closed state (Lu et al., 2002). Assuming that the voltage sensors S4 are coupled to the gate in Shaker channels via an obligatory (one S4 in the down state is enough to keep the channel closed) rather than an allosteric mechanism, they proposed a mechanical lever model in which S4S5_L_ are pushing the S6 gate in a closed conformation at negative voltages. The crystal structure corresponding to the open state of the related vertebrate Kv1.2 (below) is consistent with this mechanism in which all the S4 segments have to be in an “up” state to allow pore opening.

In other functional studies, Shaker mutations in S4S5_L_ and S6_T_ were shown to have a dramatic effect on the slow component of the off-gating current. Together with the fact that closing the gate impacts on gating charge return, this has been interpreted as the S4S5_L_ and S6_T_ interaction allosterically keeping S4 in the “up” position and stabilizing the open state (Batulan et al., 2010). The same group identified other Shaker mutations in S4S5_L_ and S6_T_ that completely uncouple S4 movement from pore opening. They used the mutations to show that the pore domain exerts a mechanical load onto the voltage sensors. Indeed, these mutations lead the voltage sensors to be activated at more negative potentials (they are more free to move), and relieve the mode shift of the voltage sensor, that they interpret as a stabilization of the open state directly impacting the S4 movement (Haddad and Blunck, 2011).

Altogether, these data support both the specificity and the strength of interaction between S4S5_L_ and S6_T_, consistent with the mechanical lever mechanism, but in a more complex manner, with potentially state-dependent S4S5_L_ and S6_T_ interactions stabilizing the closed (Lu et al., 2002) and the open (Batulan et al., 2010) states. Of note, the critical role of S4S5_L_ and S6_T_ interaction in channel open state stabilization has been recently illustrated using high speed molecular dynamics simulation (Jensen et al., 2012). Altogether these data, suggest a mechanism more complex than a pure electromechanical coupling.

###### Kv1.2

The Kv1.2 channel is a Shaker-like voltage-gated potassium channel expressed in mammalian neurons and involved in the regulation of pre- and post-synaptic membrane excitability. The interaction between the S4–S5 linker and the S6 segment was observed in the crystal structure of Kv1.2 in the open state (Long et al., 2005), confirming the electromechanical coupling between the voltage sensor movement and the pore, as suggested previously by Lu and co-workers in Shaker channels. The S4 were suggested to perform mechanical work on the pore of Kv1.2 through the S4–S5 linkers, which are positioned to constrict or dilate the S6 inner helices of the pore (Long et al., 2005). A prediction of the channel closed state was built based on the hypothesis of a permanent coupling. In this configuration where the S6 helix is presented as a “receptor” of S4–S5 linker, it is easy to understand why its sequence on K_v_ channels is quite conserved: Pro-X-Pro, where X is any amino-acid (Shaker-like K_v_ channels), or Gly (other K_v_ channels) in the corresponding region. This structure allows bending of the S6 helix in order to form the correct interaction with the S4–S5 linker helix. However, the absence of a structure of K_v_ channels in the closed state prevents from determining the exact molecular nature of the voltage-dependent gate closure. Moreover, the structure of Kv1.2 may not completely correspond to the functional open-activated state, especially for the position of S4 relative to the pore, since this structure is incompatible with the proximity of first S4 arginine R294 and a pore domain residue, A351 (Lewis et al., 2008). Such proximity between R294 and A351 was probed by the generation of a high affinity binding site of Zn^2+^ or Cd^2+^ when the residues were mutated to histidine. As discussed above for cross-linking experiments trying to estimate the S4 position and movement, introduction of the Zn^2+^ or Cd^2+^ high affinity binding site may also capture the channel in a non-native state.

Only the combination of experimental and *in silico* approaches, and the multiplication of channel structures will help understanding the molecular details of the channel voltage dependency. For instance, a recent crystal structure obtained from a prokaryotic voltage-gated sodium channel (structurally similar to eukaryotic voltage-gated K^+^ channels) supports the idea of a transient and quite labile coupling between S4S5_L_ and the S6_T_ (Figure 1B). Indeed, we can observe in this structure that the voltage sensors S4 are in their activated position even though the pore is closed (Payandeh et al., 2011), and this corresponds to a decreased interaction of S4S5_L_ with S6_T_.

#### Model 2: ligand/receptor model (Figure 1B)

##### KCNQ1

It is now admitted that the VSD-pore coupling is mediated by the interaction between S4S5_L_ and S6_T_. Several works on Shaker and Kv1.2 channels (above) suggest that the nature of this interaction is a strong coupling of the pore opening with voltage sensor movement. But in other channels, the interaction between S4S5_L_ and S6_T_ may be state-dependent, and leads to stabilization of the channel in the open or closed state. Forcing the interaction between S4S5_L_ and S6_T_ seems to stabilize hERG channels in a closed conformation (Ferrer et al., 2006). One interpretation can be that S4S5_L_ is the equivalent of a ligand, able to bind to S6_T_ and to stabilize the channel in a closed state. Upon depolarization, S4 drags the S4S5_L_ ligand away from its receptor, allowing the channel to open (Figure 1B). To test this hypothesis on KCNQ1, we designed peptides identical to S4S5_L_ (the “ligand”) and S6_T_ (the “receptor”) based on sequence alignment with Shaker, in which interacting areas in the S4S5_L_ and the S6_T_ were suggested (Lu et al., 2001, 2002). KCNQ1 coassembles with the β-subunit KCNE1 to form the channel responsible for the cardiac slowly activating delayed rectifier current, *I*_Ks_. In COS-7 cells transfected with the cardiac KCNE1-KCNQ1 channel complex and the S4S5_L_ or S6_T_ mimicking peptides, we found that co-expression of S4S5_L_ peptides (“ligand” or inhibitory peptides) and the channel resulted in a reduction of the voltage-dependent potassium currents. In contrast, S6_T_ peptides (“receptor” or decoy peptides) up-regulated channels activity, by competing with the endogenous S6_T_ and decreasing the inhibitory effect of the endogenous S4S5_L_ binding to the endogenous S6_T_ (Choveau et al., 2011). This confirms that S4S5_L_ can be compared to a ligand that locks channels in the closed state by interacting with its receptor, S6_T_. The specificity of the S4S5_L_/S6_T_ interaction was confirmed by mutating the partners. Previous mutagenesis studies in KCNQ1 channels identified mutations in S4S5_L_ (V254A) and in S6_T_ (L353A) that prevent the channels from closing completely at hyperpolarizing potentials (Boulet et al., 2007; Labro et al., 2011), consistent with a decrease in the S4S5_L_–S6_T_ interaction. Based on these results, introduction of V254A in S4S5_L_ peptide or L353A mutations in S6_T_ peptide should disrupt the channel-peptide interaction and thus abolish their respective effect on the K^+^ current. Mutant peptides have indeed no effect on KCNQ1 function (Choveau et al., 2011). To further demonstrate the specificity of the peptides-KCNQ1 interaction, a couple of mutations were tested both on the peptides and on the channel. In the KCNQ1 channel, introduction of L353A mutation located in S6_T_ leads to an instantaneous current component, that is abolished by the introduction of V254L mutation located in S4S5_L_ (Labro et al., 2011). The increased side chain volume induced by V254L substitution is probably compensating for the decreased side chain volume induced by the L353A one. We hypothesized (i) that the incomplete L353A channel closure was due to a low binding affinity of the endogenous WT ligand (S4S5_L_) to its L353A mutated S6_T_ receptor and (ii) a restored binding affinity of the endogenous V254L mutated ligand (S4S5_L_) to the mutated S6_T_ receptor. To confirm this, we showed that the WT S4S5_L_ peptide has indeed no effect on the L353A KCNQ1 channel, whereas the mutant S4S5_L_ peptide (V254L) has an effect on this L353A KCNQ1 channel (Choveau et al., 2011). Altogether, our results are consistent with a ligand/receptor mechanism in which S4S5_L_ acts as a ligand that binds to its receptor, S6_T_, stabilizing the pore in a closed conformation. May this ligand/receptor mechanism be applied to other voltage-gated channels?

##### Human ether-a-go-go related gene

The hERG encodes the voltage-gated potassium channel underlying the cardiac delayed rectifier current, *I*_Kr_, participating in the repolarization phase of cardiac action potential (Curran et al., 1995; Sanguinetti et al., 1995; Trudeau et al., 1995). hERG channel structure is similar to that of Shaker-like voltage-gated channels (Warmke and Ganetzky, 1994), possessing six (S1–S6) TMDs that comprise voltage sensor (S1–S4) and ion conduction pore (S5–S6) region. Despite this similarity, hERG channels behave very differently from Shaker-like channels: hERG activation and deactivation gating kinetics are much slower, whereas inactivation and the recovery from inactivation are rapid and intrinsically voltage-dependent (Smith et al., 1996; Sanguinetti and Tristani-Firouzi, 2006). Similarly to KCNQ1, the proximity between the S4S5_L_ and S6_T_ in the closed state was suggested by mutagenesis of these regions (Tristani-Firouzi et al., 2002). Most importantly, introducing cysteines in both S4S5_L_ and S6_T_ led to a current decrease in an oxidizing environment, and predominantly at a negative holding potential. This potential-dependent channel locking in the closed state is consistent with the formation of a disulfide bond between the cysteines introduced in S4S5_L_ and S6_T_ (Ferrer et al., 2006), and suggest that S4S5_L_ binding to S6_T_ locks the channel closed. This is in accordance with the ligand-receptor model underlying the voltage dependency of hERG channel activity. In the WT channel, interaction between S4S5_L_ and S6_T_ occurs via specific amino-acids since a point mutation (D540K) located in S4S5_L_ (Sanguinetti and Xu, 1999) fundamentally alters the gating properties of hERG channels and these changes are prevented by additional point mutations (R665A, R665Q, or R665D) located in S6_T_ (Tristani-Firouzi et al., 2002). The demonstrated specificity of amino-acids interaction further supports the S4S5_L_ ligand and S6_T_ receptor model. A companion review (Cheng and Claydon, 2012) in the present Research Topic suggests that the sequence of the S4S5_L_ may be partly responsible for the slow activation kinetics of hERG channels.

##### HCN and KAT1

The hyperpolarization-activated, cyclic-nucleotide-gated (HCN) channels represent a family of four members (HCN1-4) that carry *I*_f_ (“f” for “funny”) or *I*_h_ (“h” for “hyperpolarization”) currents (DiFrancesco, 1981). Sequence analysis revealed that the primary structure of HCN channels is similar to that of voltage-gated potassium channels, i.e., six TMDs (S1–S6), including the positively charged voltage sensor S4 and the ion-conducting pore between S5 and S6. Ionic currents through HCN channels modulate the intrinsic electrical activity in the heart (DiFrancesco et al., 1979; DiFrancesco, 1993) and in a variety of neurons (Pape, 1996). Intriguingly, these non-specific cation channels are activated upon cell membrane hyperpolarization, contrarily to the classical depolarization-activated ion channels.

How can this difference in the gating behavior be explained? Two competing models have been proposed. The first model proposes that HCN channels are in an inactivated state when the membrane is depolarized and that its hyperpolarization induces channels to recover from inactivation and enter into an open state (Miller and Aldrich, 1996; Gauss et al., 1998). The second suggests that HCN channels gating is opposite to the one of K_v_ channels. In other words, membrane depolarization induces HCN channels deactivation whereas membrane hyperpolarization results in channel activation. Uncovering hyperpolarization-induced inactivation in KAT1, a six-segment potassium channel cloned from the higher plant *Arabidopsis* and having similar gating characteristics as HCN, has provided an argument that favors the second model for hyperpolarization-dependent activation of HCN channels (Moroni et al., 2000).

Alanine-scanning mutagenesis in HCN2 channel identified three S4S5_L_ residues playing a major role in the S6 gate stabilization in the closed state (Chen et al., 2001), consistent with the “ligand/receptor” model of voltage dependency described in KCNQ1 and hERG. However, this does not explain in a straightforward way the reversed voltage dependency of the channel compared to other voltage-gated channels. A possible explanation would be that a specific S4S5_L_–S6_T_ interaction also favors an open state (in mirror to such interaction favoring a closed state in KCNQ1 or hERG channels). Using a cysteine cross-linking approach, a study showed that forced interaction between the S4S5_L_ (F359C) and the C-terminus, downstream to S6 (K482C), leads to a constrained and unnatural opening of spHCN1 channel (Prole and Yellen, 2006). Using a homology modeling approach, another study on KAT1 suggested that channel closure occurs via an electrostatic repulsion between S4S5_L_ (R190 and R197) and S6_T_ (R307 and R310) while the channel opening occurs when S4S5_L_ is rotating, allowing an electrostatic interaction between D188 in S4S5_L_ and R307, R310 in S6_T_ (Grabe et al., 2007). Again, all these studies are in good agreement with a ligand/receptor model of voltage dependency.

##### Kv4.2

Kv4.2 channel belongs to the family of voltage-gated potassium channels related to the *Shal* gene of *Drosophila* (Kv4 channels). These channels mediate a subthreshold-activating current (*I*_SA_) that controls dendritic excitation and the backpropagation of neuronal action potentials (Hoffman et al., 1997). These Kv4 channels share structural motifs that are conserved in Shaker-like K_v_ channels, including the positively charged S4 voltage sensor, the TTXGYGD signature sequence in the selectivity filter, and the Pro-X-Pro motif in the S6 segment. One specificity of these channels, as compared to Shaker-like channels, is their significant closed-state inactivation induced by small depolarization (Jerng et al., 2004) and a fast voltage-dependent recovery from inactivation (tens to hundreds of milliseconds). Using functional and modeling approaches, it was demonstrated that this closed-state inactivation is strongly linked to the S4-charge immobilization in Kv4.2 channels, suggesting that the functional availability of Kv4.2 channels is directly regulated by the voltage sensors (Dougherty et al., 2008). Another study based on structural modeling and alanine-scanning, demonstrated that this voltage-dependent regulation involves a dynamic coupling between the S4S5_L_ and S6_T_. This dynamic coupling mediates both transient activation and closed-state inactivation in Kv4.2 channels (Barghaan and Bähring, 2009). While interaction between S4S5_L_ and S6_T_ is necessary for channel activation, the Kv4 inactivation process would result from a destabilization of this interaction. This is detailed in another review (Bähring, 2012) of the present Research Topic. A model of labile coupling might thus be applied to Kv4.2 channels the same way as for KCNQ1, hERG, HCN, or KAT1 channels.

##### Na_v_ and Ca_v_ channels

Voltage-gated Na^+^ and Ca^2+^ channels (Na_v_ and Ca_v_, respectively), are fused tetrameric subunits with the same structural organization as proper tetrameric K_v_ channels. Indeed, Na_v_ and Ca_v_ subunits contain four homologous but not identical domains, each including six transmembrane segments (S1–S6), a voltage sensor domain with a positively charged S4 segment and a pore region formed by the association of S5 and S6 segments.

Since the voltage-dependent activity of Na^+^ and Ca^2+^ channels is mediated by the S4 movements in response to membrane potential variation (Yang and Horn, 1995; Hu et al., 2003) like voltage-gated potassium channels, we hypothesize that the ligand-receptor mechanism we demonstrated for KCNQ1 (Choveau et al., 2011; Labro et al., 2011) may be applied to Na^+^ and Ca^2+^ channels. The recent crystal structure of the prokaryotic one-domain voltage-gated sodium channel is consistent with our hypothesis since it can be observed that the channel gate (S6) is closed while the S4 segments are in the “up” position (Payandeh et al., 2011). Moreover, in this pre-open configuration (or pre-locked configuration if we consider the open to close pathway), the interaction surface between S4S5_L_ and S6_T_ is reduced as compared to the Kv1.2 channels structure (Payandeh et al., 2011). These observations support the model of a spontaneously opening and closing pore (McCusker et al., 2011; Shaya et al., 2011) with S4S5_L_ locking the channel in a closed state when the membrane is polarized (Figure 1B). It will be interesting to confirm if this model also applies to Na^+^ and Ca^2+^ channels, using the approach of exogenous peptides mimicking S4S5_L_ or S6_T_, as used in Choveau et al. (2011).

### Impaired S4–S5 and S6 interaction underlies human diseases

As developed earlier, it is broadly accepted that the interaction between S4S5_L_ and S6_T_ is extremely important for voltage-gated ion channels function (activation, deactivation, and inactivation). For that reason, disruption of such interaction may have dramatic physiological effects, and lead to certain forms of disease.

Both cardiac and neurological disorders have been linked to impaired S4–S5_L_ and S6_T_ interactions in K_v_ channels. For instance, many mutations of the KCNQ1 channels lead to the LQT, a cardiac disease characterized by prolonged ventricular repolarization, arrhythmias, and sudden death. Interestingly, looking specifically at the S4S5_L_, it was shown that LQT1 mutations (type 1 LQT, associated with mutations in KCNQ1) are clustered on the one side of the S4S5_L_ α-helix structure, that is putatively responsible for interactions with the S6_T_ region (Boulet et al., 2007; Labro et al., 2011), while several LQT1 mutations are also localized in the interacting S6_T_ region (http://www.fsm.it/cardmoc/), comforting in the opinion that the interaction of S4S5_L_ with S6_T_ is physiologically crucial for a proper heart function. Unfortunately, specific studies that would directly relate the importance of this interaction with disease are still lacking. However, in order to confirm that the ligand/receptor model (Figure 1B) fits well with the KCNQ1-E1 complex behavior, we used an atrial fibrillation mutant, S140G, that was shown to deactivate extremely slowly, and thus that presents almost no voltage dependence in the −80 to +80 mV range (Chen et al., 2003; Restier et al., 2008). Interestingly, while “S6_T_/activator peptides” clearly affect WT KCNQ1-KCNE1 channels, no effect was observed on the S140G-E1 complex. Conversely, “S4S5_L_/inhibitory peptides” did have a dramatic blocking effect, suggesting that the endogenous S4S5_L_ of the S140G mutant channel does not reach S6_T_. Although speculative, these data suggest that in this mutant, the gain-of-function effect might be somehow related to an impaired interaction between S4S5_L_ and S6_T_ (Choveau et al., 2011) due to a stabilization of S4 in the “up” state (Restier et al., 2008).

On the other hand, the pathological effect of a Kv1.1 channel mutation is consistent with the mechanical lever model of Kv1 channels (Figure 1A): the observation that a mutation located in the S4S5_L_ prevents Kv1.1 open state stabilization led to the conclusion that disrupted S4S5_L_ and S6 interactions underlie one type of episodic ataxia disease, in direct support of the mechanical lever model (Batulan et al., 2010).

Recently, it was proven that S4S5_L_ and S6 regions of the voltage-gated calcium channel Cav2.3 are coupled during the activation process (Wall-Lacelle et al., 2011). Since Ca_v_ channels are involved in several pathologies, including episodic ataxia, familial hemiplegic migraine, idiopathic generalized epilepsy (Adams and Snutch, 2007), one can easily imagine that an impaired S4S5_L_–S6_T_ interaction in these channels might also underlie diseases, knowing that mutations in patients have been found in those critical regions (Adams and Snutch, 2007; Pietrobon, 2007).

## Part 2: Modulation of Voltage-Gated Channels by PIP_2_

### PIP_2_ regulates several voltage-gated channels

#### KCNQ1 channels

##### Effect of PIP_2_ on *I*_Ks_ currents

**Figure 2:**
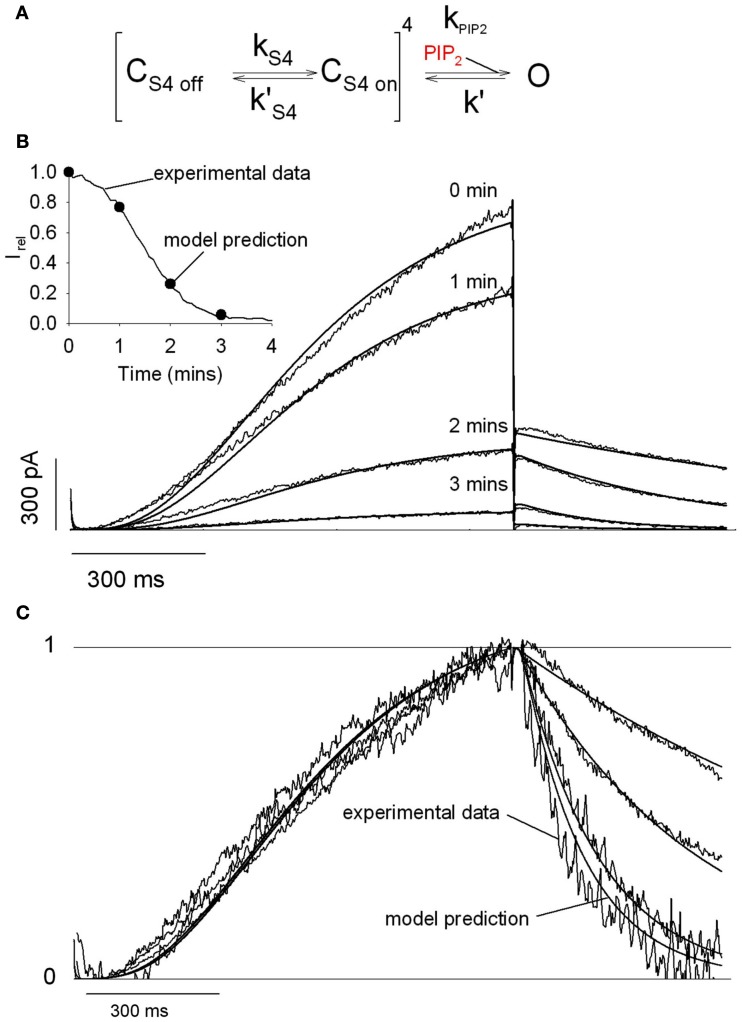
**A model based on the stabilization of the open state by PIP2 recapitulates the characteristics of the KCNQ1/KCNE1 currents**. **(A)** During activation, $k_{s4}^{’}$ is negligible whereas *k*_S4_ = 3.56/s, and during deactivation $k_{\text{s}4}^{’}=7.47/\text{s}$ whereas *k*_S4_ is negligible. In this model, PIP_2_ only affects the transition from a closed state to an open state when the four voltage sensors are in the permissive state (*C*_S4 on_). Thus, during simulated rundown, only *k*_PIP2_ varies (*k*′ = 87.3/s). **(B)** Experimental traces were superimposed with the simulated current (solid lines). *k*_PIP2_ was fixed to 592.74, 176.43, 25.84, and 4.53/s (simulating PIP_2_ level decrease) to best fit the decrease in current amplitude during rundown, as shown in the inset. Inset: simulated (circles) and observed current (solid line) amplitudes as a function of time after patch excision. **(C)** Traces in **(B)** were normalized to compare the observed and simulated kinetics of activation and deactivation. From Loussouarn et al. (2003).

Phosphatidylinositol 4,5-bisphosphate (PIP_2_) is a minor acidic membrane lipid found primarily in the inner leaflet of the plasma membrane. PIP_2_ was first described as the precursor of the second messengers inositol 1,4,5-trisphosphate (IP_3_) and diacylglycerol (DAG) when cleaved by receptor-activated phospholipase C (PLC; Berridge, 1981). It was realized much later that plasma membrane PIP_2_ is not simply a precursor, but also a signaling molecule in its own right (reviewed in Logothetis et al., 2010). As also demonstrated for a wide variety of ion channels and transporters (Gamper and Shapiro, 2007; Suh and Hille, 2008; Logothetis et al., 2010), we showed that PIP_2_ is a necessary cofactor for KCNQ1 channel activity (Loussouarn et al., 2003). It regulates KCNQ1 channel function by stabilizing its open conformation, leading to increased current amplitude, slower deactivation kinetics, and a negative shift in the steady-state activation curve. Such PIP_2_ effect was described by a kinetic model in which only the final concerted step toward opening was affected by PIP_2_ levels (Figure 2). In this model, when the membrane is depolarized, the movement of the four voltage sensors in the upward direction is rate limiting, making activation kinetics PIP_2_-independent. But, when the membrane is repolarized, the transition of the concerted pore closing becomes rate limiting, making deactivation kinetics PIP_2_-dependent. Other KCNQ channels are also PIP_2_ sensitive, like the KCNQ2/KCNQ3 complex responsible for the neuronal M-current (cf. below). It is interesting to note that for this channel complex, the biophysical parameters do not seem to vary as PIP_2_ levels vary (Shapiro et al., 2000), and more specifically the deactivation kinetics (Zhang et al., 2010). It is possible for those channels, that the concerted pore closing is not rate limiting, making deactivation kinetics PIP_2_-independent. In KCNQ4, similar kinetics of “OFF” gating current and ion current deactivation are consistent with this hypothesis (Miceli et al., 2012).

The KCNQ1/KCNE1 kinetic model shares similarities with the one of Kir6.2/SUR1 channel (Enkvetchakul et al., 2000) suggesting similar effects of PIP_2_ on six-domain and on two-domain channels. Furthermore, similarly to several inwardly rectifying K^+^ (K_ir_) channels, ROMK, GIRK, and IRK (Huang et al., 1998), direct interaction of PIP_2_ with a cluster of basic residues located in the C-terminus close to S6 was recently shown in KCNQ1 channel (Thomas et al., 2011). This functional homology may give some insights on the nature of PIP_2_ regulation of KCNQ1/KCNE1 channels. From the crystal structure of a GIRK channel, Whorton and Mackinnon showed that PIP_2_ molecules lie at the interface between the TMD and the CTD (TMD-CTD) and are coordinated by several positively charged residues Lys64, Lys194, Lys199, and Lys200 (Whorton and Mackinnon, 2011). PIP_2_ is suggested to couple the G-loop gate (open by GTP binding) and the inner helix gate. But even in the absence of GTP, it allows the outer and interfacial helices to slightly shift downward and outward, and the inner helices to slightly rotate. Even if the motion of the inner helices is not sufficient to open the pore by itself, it shows that PIP_2_ binding can lead to the inner helices rearrangements. For KCNQ1, PIP_2_ binding to the cluster of basic residues located just after S6 (Thomas et al., 2011) may lead to the stabilization of the inner helices in an open state. In another recent crystallographic study, Hansen et al. (2011) showed that PIP_2_ mediates docking of the whole CTD to the TMD and subsequent opening of the inner helix gate of Kir2.2. Thereby, we can speculate that KCNQ1 CTD could interact with the membrane, via interactions with PIP_2_. This idea is supported by our previous work showing that substitutions of arginines located at the C-terminus of KCNQ1 channels (R539 and R555, cf. below) decrease the channel-PIP_2_ sensitivity. However, crystallographic studies must be done to confirm this hypothesis.

##### Impact of KCNE1 subunits on PIP_2_ sensitivity of *I*_Ks_

Although KCNQ1 is a voltage-gated channel on its own, KCNE1 leads to changes in the current properties: it increases the amplitude, shifts the voltage dependence of activation toward more positive potentials, slows activation and deactivation kinetics, and suppresses inactivation (Barhanin et al., 1996; Sanguinetti et al., 1996). More recently, it was shown that KCNE1 alters the function of *I*_Ks_ by modulating the interaction between PIP_2_ and the KCNQ1/KCNE1 complex (Li et al., 2011). It is established that the interaction between proteins and PIP_2_ is often based on interaction between basic residues with the negative charges of PIP_2_ (Suh and Hille, 2008). In light of this, Li et al. (2011) individually mutated 11 basic residues located in the cytosolic C-terminus of KCNE1 to identify key structural determinants contributing to *I*_Ks_ regulation by PIP_2_. To do this, they studied for each mutant the gradual decrease of KCNQ1/KCNE1 channel activity (“rundown”) observed right after excision in the inside-out configuration of the patch-clamp technique, patch excision provoking a decrease in membrane PIP_2_ levels. In their study, Li et al. (2011) demonstrated that KCNE1 increases the PIP_2_ sensitivity of *I*_Ks_. More specifically, they identified 4 basic residues (R67, K69, K70, and H73) in KCNE1 that seem to play a critical role in this PIP_2_ sensitivity. They showed that neutralization of these basic residues abolished the delay before rundown that is specifically observed when KCNE1 is co-expressed with KCNQ1, and significantly reduced the time constant of rundown. From a structure obtained by a NMR approach, it appears that these four residues are located on an α-helix, following the TMD (Kang et al., 2008). Kang et al. suggested that the C-terminal end of KCNE1 sits near S4S5_L_ and S6_T_, which may explain the changes exerted by KCNE1 on the gating of KCNQ1 (see Part 1: Role of the S4–S5 Linker in Channel Voltage Dependency). Moreover, basic residues in the S4S5_L_ and in the proximal C-terminus of KCNQ1 have been shown to interact with PIP_2_ (Park et al., 2005; Thomas et al., 2011), suggesting that PIP_2_ and KCNE1 modulate *I*_Ks_ through interaction with the same region of KCNQ1. Thus, PIP_2_ interacts with amino-acids in the KCNQ1/KCNE1 channel complex, and its capacity to modulate *I*_Ks_ is regulated by KCNE1, through mechanisms that remain to be clearly identified by crystallographic approach.

##### Impact of PKA and PKC on PIP_2_ sensitivity of *I*_Ks_

Neurotransmitter and hormone receptor stimulations activate different signaling pathways that adjust the protein phosphorylation status. Among others, Gq/G11-protein coupled receptors, like muscarinic acetylcholine (ACh) receptors (M1), stimulate the PLC which hydrolyzes PIP_2_ (Berridge, 1981) as explained above. The DAG produced by PIP_2_ hydrolysis activates protein kinase C (PKC), which has been suggested to regulate *I*_Ks_ channels. Matavel and Lopes (2009) showed that Gq-coupled receptors regulate *I*_Ks_ in a biphasic manner: (i) downstream activation of PLC leads to PIP_2_ depletion and underlines channel inhibition and (ii) PKC-mediated phosphorylation is responsible for the activation phase.

**Figure 3:**
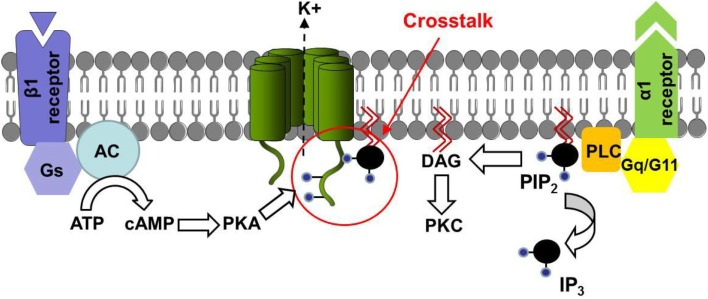
**Model of the regulation of channel-PIP2 interactions by PKA**. β1-adrenergic receptor regulates channel-PIP_2_ interaction through PKA phosphorylation, and simultaneously regulates Gq/G11-protein coupled modulation of the channel. Lopes et al. showed that this model is transposable to KCNQ1 and TREK1 channels. Adapted from Lopes et al. (2007).

Protein kinase A (PKA) is another well-characterized kinase that regulates *I*_Ks_ through receptor-activated signaling pathways (Walsh and Kass, 1988; Marx et al., 2002). Stimulation of the β1-adrenergic receptor leads to activation of adenylyl cyclase (AC) that catalyzes the conversion of ATP to cAMP and activates PKA. This β-adrenergic stimulation activates KCNQ1 via direct phosphorylation by PKA. More recently, Lopes et al. (2007) showed a crosstalk between KCNQ1 phosphorylation by PKA and its regulation by G-proteins of the Gq/G11 family. This study demonstrated that ACh inhibition of KCNQ1/KCNE1 currents in injected *Xenopus laevis* oocytes was lower in activated-PKA conditions and higher in inhibited-PKA conditions as compared to control. Furthermore, invalidation of the KCNQ1 S92 consensus phosphorylation site completely abolished the PKA effect on M1 inhibition of KCNQ1/KCNE1 currents. These results suggest that direct PKA phosphorylation of KCNQ1 is responsible for the PKA modulation of the observed PLC-dependent inhibition. A direct effect of PKA phosphorylation on channel regulation by PIP_2_ was suggested by the use of wortmannin, which blocks the PI4-kinase intervening in the PIP_2_ synthesis. PKA modulation of wortmannin inhibition was similar to the PKA modulation of M1 inhibition of KCNQ1. All these results suggest that the KCNQ1 sensitivity to PIP_2_ is modulated by PKA (Figure 3).

More recently, Matavel et al. (2010) gave some new insights on KCNQ1 regulation by PKA and PKC. They tested four point mutations of putative PIP_2_ interaction sites of the channel (R174C, R243C, R366Q, and R555C) and observed that mutations located in the proximal and distal C-terminus (R366Q and R555C, respectively), enhance the channel sensitivity to variations of membrane PIP_2_ level, suggesting a decrease in the apparent affinity of these mutant channels to PIP_2_. This was not the case for two mutations located in the S2–S3 loop (R174C) and in the S4S5_L_ (R243C). For the latter, this is in contradiction with the enhanced sensitivity to PIP_2_ level variation observed by Park et al. (2005) for the R243H mutant. Such discrepancy can be explained by the difference in the nature of the substituted amino-acid (cysteine in one case and histidine in the other) or by differences in experimental conditions (whole-cell configuration on oocytes and giant-patch configuration on COS-7 cells, respectively). Furthermore, Matavel et al. showed that R174C and R243C mutants exhibited an impaired activation by both PKA and PKC, whereas C-terminal KCNQ1 mutants presented an increased activation. Thus, for R366Q and R555C mutant channels, regulation of the channel by PIP_2_ was potentiated, suggesting that PKA and PKC activate the channel by strengthening KCNQ1 interactions with PIP_2_.

#### Other KCNQ channels

Five members have been identified in the KCNQ channel family (KCNQ1–5), each with a specific tissue distribution. In the heart, intestine, and inner ear, KCNQ1 subunits, assembling with auxiliary KCNE subunits, are important for repolarization and K^+^ transport (Barhanin et al., 1996; Sanguinetti et al., 1996; Neyroud et al., 1997; Wang et al., 1998b). KCNQ2, KCNQ3, and KCNQ5 participate to “M-type” K^+^ currents in a variety of neurons (Lerche et al., 2000; Schroeder et al., 2000; Cooper et al., 2001; Roche et al., 2002; Shah et al., 2002) and play a dominant role in regulating neuronal excitability (Jones et al., 1995; Cooper et al., 2001). KCNQ4 primarily localizes to the inner ear (Kubisch et al., 1999). Zhang et al. (2003) studied the PIP_2_ dependency of all KCNQ family members. They used various approaches for homomeric KCNQ2 and heteromeric KCNQ2/KCNQ3 channels and showed that PIP_2_ application in inside-out macropatches leads to an increase in channel activity, even after an almost complete rundown. Following channel reactivation by PIP_2_, they observed that application of polylysine, which was described to act as a PIP_2_ scavenger (Lopes et al., 2002; Rohács et al., 2002), results in fast and complete block of the current. Application of PIP_2_ antibody to the internal surface of inside-out macropatches also suppresses the current. KCNQ1/KCNE1, KCNQ4, and KCNQ5 channels are also reactivated by PIP_2_ after inhibition by polylysine, showing that all KCNQ family members are PIP_2_ sensitive.

Similar to KCNQ1 (Loussouarn et al., 2003), PIP_2_ may increase the current via a stabilization of the open state of KCNQ2–4 channels (Li et al., 2005). In their study, Li et al. (2011) showed that the maximal single-channel open probability (*P*_o_) of KCNQ2–KCNQ4 and specifically KCNQ2/3 channels is highly governed by diC8-PIP_2_ concentration. Furthermore, they observed a strong increase in maximal channel open probability (*P*_o_) of KCNQ2/3 and KCNQ2 in cell-attached patches from cells overexpressing PI5-kinase, which has been shown to increase membrane PIP_2_ (Bender et al., 2002; Winks et al., 2005). Conversely, a decrease in free membrane PIP_2_ induced by muscarinic stimulation strongly lowers channel *P*_o_. The apparent affinity of the channels for diC8-PIP_2_ is strongly different and parallels the differential maximal *P*_o_ in cell-attached patches, suggesting that *P*_o_ of channels is mainly governed by their sensitivity to membrane PIP_2_ (Li et al., 2005). Although not sufficient to nail down the point, these experiments are consistent with PIP_2_ stabilizing the open state of all KCNQ channels.

In addition to PIP_2_, several kinds of phosphoinositides but also other phospholipids are present in the plasma membrane and are capable of regulating the “M-type” K^+^ current (Telezhkin et al., 2012). However, the fact that the current decreases when using tools that specifically decrease PIP_2_ (Suh et al., 2006; Lindner et al., 2011) plus consideration of the concentration for half activation for the different phospholipids and their abundance in the membrane suggest a predominant role of PIP_2_ for the regulation of KCNQ channels (Telezhkin et al., 2012).

#### Human ether-a-go-go related gene

The hERG or KCNH2 encodes the pore-forming subunit of the channel that is responsible for the rapid delayed rectifier K^+^ current, *I*_Kr_, in cardiac cells and several other cell types (cf. Part 1: Role of the S4–S5 Linker in Channel Voltage Dependency). This was the first voltage-gated ion channel described to be sensitive to PIP_2_ (Bian et al., 2001). Consistent with this PIP_2_ sensitivity, the muscarinic receptor M1, which stimulates enzymatic hydrolysis of PIP_2_ by PLC, has been shown to suppress rat ERG currents in a heterologous system (Hirdes et al., 2004). As opposed to KCNQ1/KCNE1 (Loussouarn et al., 2003), Bian et al. (2001) showed that PIP_2_ addition on hERG channel led to an accelerated activation with no effect on deactivation. But more recently, we observed that PIP_2_ effects on hERG are very close to those observed on KCNQ1/KCNE1: increased current, slowed deactivation, and no effect on activation kinetics (Rodriguez et al., 2010). This difference could be due to the use of divergent patch-clamp configurations in these studies: whole-cell in Bian et al. versus inside-out in our study. Furthermore, as for KCNQ1/KCNE1 channel complex, a kinetic model showed that PIP_2_ effects on hERG can be explained by modifying the late transition rates only, corresponding to pore opening. In addition, we observed that hERG channels present a PIP_2_ sensitivity similar to KCNQ1/KCNE1, estimated by (i) polylysine-induced rundown kinetics, (ii) PIP_2_ induced run-up kinetics, and (iii) sensitivity to intracellular Mg^2+^, which is known to screen the PIP_2_ negative charges. All these data support the idea that hERG and KCNQ1/KCNE1 channels have a similar affinity to PIP_2_. However, the experiments we performed also showed the persistence of a fraction of hERG current at low PIP_2_ levels, which may underlie differences in response to physiological decrease in membrane PIP_2_ levels.

#### Other voltage-sensitive channels

In addition to the delayed rectifiers KCNQ1 and hERG, other voltage-gated channels are regulated by PIP_2_: the voltage-gated Ca^2+^ channels (Ca_v_) channels (Wu et al., 2002), HCN channels (Pian et al., 2006), and also K_v_ channels (Oliver et al., 2004). At least for Ca_v_ channels, accessory subunits can regulate the modulation of the current by PIP_2_, similar as the β-subunit KCNE1 modulating the PIP_2_ sensitivity of KCNQ1 (Suh et al., 2012). Another article of this Frontiers Research topic is focusing on the effect of PIP_2_ on these channels (Menchaca et al., under revision).

### Implication of PIP_2_ in signaling pathways

#### Depletion of PIP_2_ by activation of the Gq signaling pathway

Many studies have investigated the role of PIP_2_ in the regulation of voltage-gated KCNQ channels activity. Recovery of KCNQ2/KCNQ3 current following muscarinic stimulation requires re-synthesis of PIP_2_ (Suh and Hille, 2002) and channels activity decreases quickly upon patch excision but is restored upon cytoplasmic addition of PIP_2_ (Zhang et al., 2003). In addition, fluorescent PIP_2_-sensitive probes showed close correlation between PIP_2_ hydrolysis and channel current suppression by muscarinic agonists (Winks et al., 2005). Similar effects of PIP_2_ were found for KCNQ1 channels, in recombinant systems (Loussouarn et al., 2003; Zhang et al., 2003; Matavel and Lopes, 2009). Surprisingly, one study shows the opposite effect of PIP_2_ on *I*_Ks_ in guinea-pig cardiomyocytes which would deserve a closer look (Ding et al., 2004).

**Figure 4:**
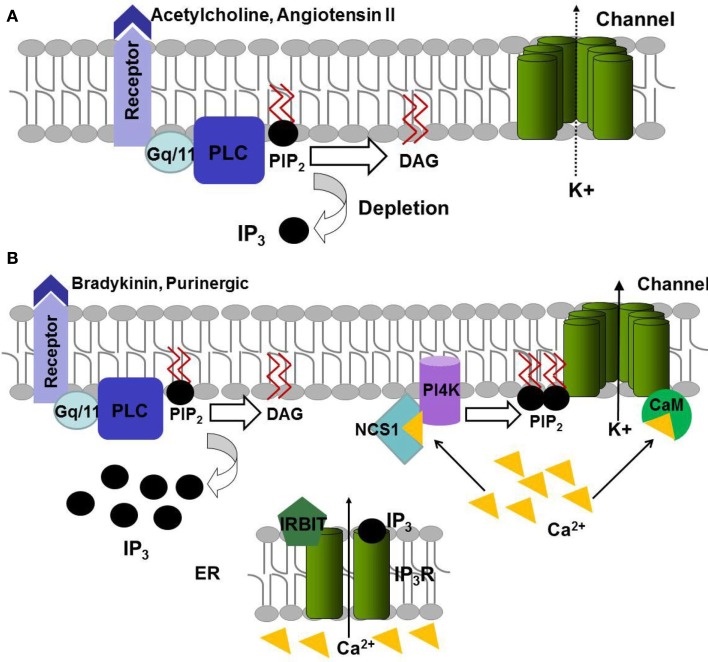
**Model of regulation of KCNQ channels function by the Gq signaling pathway**. **(A)** Activation of the PLC by ACh and angiotensin II induces the hydrolysis of PIP_2_ to DAG and IP3. **(B)** Activation of bradykinin and purinergic receptors leads to depletion of PIP_2_ by PLC but also its re-synthesis. IP_3_ allows releasing Ca^2+^ from endoplasmic reticulum via IP_3_R receptor. The released Ca^2+^ can bind to NCS-1 that induces the synthesis of PIP_2_ via PI4K or bind to CaM and modulate the channel sensitivity to PIP_2_.

A decrease in PIP_2_ may be the major determinant for a decrease in a KCNQ current upon activation of some Gq/11-coupled receptors, but the mechanism may also be more complex for other Gq/11-coupled receptors. Regarding regulation of the M-current, two distinct pathways following PLC activation and IP_3_ and DAG production have been described (Figure 4).

The first pathway, for which the decrease in PIP_2_ is the major determinant of M-current depression, is induced by the activation of M1 muscarinic ACh and AT1 angiotensin II receptors (Zaika et al., 2006; Suh and Hille, 2007; Matavel and Lopes, 2009; Figure 4A).

The second pathway, activated by bradykinin B2 and purinergic P2Y receptors (Figure 4B), induces PIP_2_ hydrolysis, but also PIP_2_ re-synthesis preventing a decrease in PIP_2_ abundance. PIP_2_ re-synthesis is triggered by the increase of IP_3_ concentration leading to calcium release from intracellular stores (Cruzblanca et al., 1998; Bofill-Cardona et al., 2000; Delmas et al., 2002; Zaika et al., 2007). This release is modulated by a IP_3_ receptor-binding protein, IRBIT, which leaves and unmasks some IP_3_ binding sites at a high enough IP_3_ concentration, and increases the IP_3_ receptor sensitivity (Zaika et al., 2011). The released Ca^2+^ binds to the calcium sensitive neuronal calcium sensor-1 (NCS-1) that activates PI4-kinase, leading to PIP_2_ re-synthesis compensating the hydrolysis of PIP_2_ by PLC (Zaika et al., 2007). Ca^2+^ also binds to calmodulin (CaM; Gamper et al., 2005) and Ca^2+^-CaM binding to the channel might decrease the affinity of channels for PIP_2_ (Kwon et al., 2007; Sarria et al., 2011) as their putative binding modules seem to overlap (Hernandez et al., 2008). This decrease in the affinity for PIP_2_ may be the cause for current depression in the second pathway (Figure 4B).

#### Binding/unbinding of PIP_2_

##### Localization of PIP_2_-binding sites

**Figure 5:**
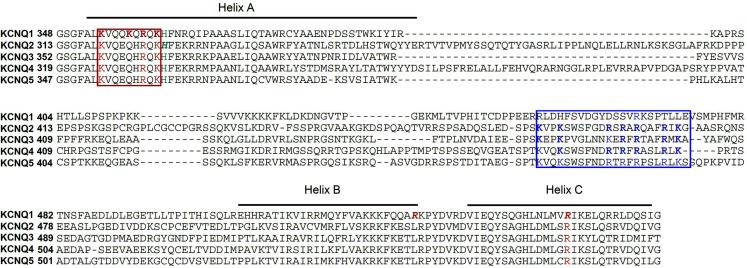
**Alignment of human KCNQ C-terminus and potential PIP2 interacting residues**. The residues in KCNQ1, framed in red and indicated in red and italic, are those identified by Thomas et al. (2011) and Park et al. (2005), respectively. The H328 residue identified in KCNQ2 is indicated in green and in italic (Zhang et al., 2003). The region identified in KCNQ2–4 by Shapiro and co-workers is framed in blue (Hernandez et al., 2008).

The location of presumed PIP_2_-binding sites and the characteristic of their motifs have been investigated in several channels. For KCNQ channels, evidence support the idea that the PIP_2_-binding site(s) is (are) located mainly within the C-terminus. For instance, the H328C mutation in helix A within the C-terminus of KCNQ2 (residue in green in Figure 5) renders channels less sensitive to PIP_2_ (Zhang et al., 2003). In addition, Shapiro and co-workers localized a cluster of basic residues within the linker connecting helices A and B in the C-terminus of KCNQ2–4 as the primary site of PIP_2_ action (Hernandez et al., 2008). Based on the crystal structure of Kir2.1, homology modeling of KCNQ2 has suggested three residues (R459, R461, and R463) to form hydrogen bonds with phosphates of the PIP_2_ head group (Hernandez et al., 2008).

Because all KCNQ channels share a common structure and are up-regulated by PIP_2_ (Loussouarn et al., 2003; Zhang et al., 2003), PIP_2_-binding site may be located at the analogous position in KCNQ1. However, a sequence alignment shows that the putative amino-acids binding to PIP_2_ identified by Shapiro and co-workers (blue frame in Figure 5) are highly conserved in KCNQ2–5 but not in KCNQ1, suggesting different PIP_2_-binding site(s) in this latter. A recent study has identified a cluster of basic residues (K354, K358, R360, and K362) in helix A of KCNQ1 as being involved in PIP_2_-binding (Thomas et al., 2011). Three of these residues are conserved in other KCNQ channels (red frame in Figure 5), suggesting a potential role of those amino-acids in PIP_2_-channel interactions in KCNQ2–4. Other residues, that are located in the S4–S5 linker (R243), downstream of the CaM binding domain (R539) and in helix C of KCNQ1 C-terminus (R555), have also been proposed to interact with PIP_2_ (Park et al., 2005). As a result, and especially for KCNQ1, PIP_2_ seems to interact with multiple parts of the channel. The crystal structures of Kir2.2 and GIRK 2, corresponding to S4–S5 linker + S5–S6 + C-terminus in KCNQ channels, illustrate such networks of interaction, and may give insights on the nature of PIP_2_ regulation of KCNQ channels, as exposed above (Hansen et al., 2011; Whorton and Mackinnon, 2011). A more precise mapping and robust structural data remain to be established in KCNQ channels to understand the underlying mechanism.

In hERG also, a PIP_2_-binding site seems to be located in the C-terminus. Deletion of a segment (883–894) in the C-terminus of hERG abolished the effects of PIP_2_ on channel amplitude and voltage dependence of activation (Bian et al., 2004). However, the exact position remains elusive.

##### Role of Ca^2+^-CaM

We described above that activation of Gq/11 signaling pathways leads to PIP_2_ depletion and consequently to decreased channel current. However, several works suggest that unbinding of PIP_2_ due to decreased affinity for KCNQ channels, rather than PIP_2_ depletion, can underlie Gq/11-mediated depression of KCNQ current (Delmas and Brown, 2005). In agreement with this, Ca^2+^-CaM binding site is very close to the putative binding site for PIP_2_: helix A and B for Ca^2+^-CaM and the A-B helix linker for PIP_2_ (Wen and Levitan, 2002; Gamper and Shapiro, 2003; Hernandez et al., 2008). This proximity indicates that occupation of this site by Ca^2+^-CaM might reduce the binding of PIP_2_ to the channel, leading to a down-regulation of channels. Consistent with this, a recent study (Sarria et al., 2011) showed an increase of the open probability by PIP_2_ of another six-segment channel, TRPM8, to be reversed by Ca^2+^-CaM. Conversely, Kwon and co-workers have found that PIP_2_ reduced Ca^2+^-CaM binding to several channels including TRPC1, TRPC5–7, and TRPV1 (Kwon et al., 2007). Interestingly, similar effects are observed in KCNQ1 and Cav1.2, supporting the idea that PIP_2_- and Ca^2+^-CaM binding sites overlap in these channels (Kwon et al., 2007). However, mechanisms by which Ca^2+^-CaM and PIP_2_ antagonize each other effects remain unclear. Does this reduction result from a direct competition or from allosteric conformational changes?

##### Do phospholipids affect the voltage sensor S4 movement?

As previously described in this review, mutagenesis studies have identified clusters of positively charged residues, mainly located in the cytosolic C-terminus of channels that may interact with the negatively charged PIP_2_. The S4 segment possesses several positively charged residues, suggesting that PIP_2_ might also affect its movement by interacting with some of these residues.

Several studies are consistent with the idea that lipids can interact with the voltage sensor and modulate its motion; although most of these studies focus on interactions in the outer leaflet (PIP_2_ is situated in the inner leaflet). Structural studies on KvAP and on a Kv1.2-Kv2.1 chimeric channel show that some residues of S4 are exposed to lipids (Lee et al., 2005; Long et al., 2007). Chimeric Kv2.1 in which the “paddle” motif (S3b and S4) is replaced by one of the paddle motifs of Nav1.4 or of the voltage-dependent phosphatase, Ci-VSP, can be used to evaluate the contribution of the paddle motif to a specific property of the voltage sensor. Hydrolysis of the outer-leaflet lipid sphingomyelin to ceramide-1-phosphate by sphingomyelinase D alters the S4 movement differently in the different chimeric channels, suggesting an interaction between outer-leaf lipids and the paddle motif (Milescu et al., 2009). The sphingomyelin phosphate that persists in ceramide-1-phosphate is critical for their interaction with S4 since sphingomyelinase C, an enzyme which removes this phosphate group, strongly reduced the gating current of Shaker and Kv1.3 (Xu et al., 2008). The importance of the phosphate group of lipids in the S4 movement has also been highlighted in KvAP channels (Schmidt et al., 2006; Zheng et al., 2011). Expression of KvAP in membranes, in which lipids have a positively charged group instead of a phosphate group, renders the channels not functional (Schmidt et al., 2006). This effect would arise from a disruption of the interaction between the arginines of the S4 segment and the phosphate groups of the membrane lipids. Consistent with this, Zheng et al., 2011) showed that the switch of the S4 from the resting to the activated conformation requires more energy in a membrane without phospholipids.

According to those studies, S4 is stabilized in the activated position by interaction with outer-leaflet phospholipids. The structure of the Kv1.2-Kv2.1 chimeric channel suggests that an inner-leaflet phospholipid may also interact with the S4–S5 linker (Long et al., 2007). We suppose that the negatively charged phosphate groups of PIP_2_ may bind to positively charged residues at the bottom of S4 or in S4–S5 linker and regulate S4 motions. However, no direct evidence exists for such an interaction.

### Impaired channel-PIP_2_ interaction underlies human diseases

As mentioned above, the importance of PIP_2_ regulation of voltage-gated ion channels is now proven and clear. Thus, one might ask how far this crucial factor affects the physiological functions of these channels. Is it limited to a biophysical/regulatory effect, or does it have major impact; for instance, can an impaired interaction with PIP_2_ lead to human disease? While this issue was partly answered for non-voltage-gated ion channels (Logothetis et al., 2010), the relationship between PIP_2_ and channelopathies implying voltage-gated ion channels is less clear, probably since the study of their regulation is more recent and less developed.

The KCNQ1-KCNE1 potassium channel complex underlies the *I*_Ks_ repolarizing cardiac current. We showed that this channel function is dependent on PIP_2_ regulation, which allows stabilization of the open state (Loussouarn et al., 2003). Importantly, we also demonstrated that residues in intracellular part of KCNQ1 channels (S4S5_L_ and C-terminus) are important for PIP_2_ regulation, and that their substitution, occurring in some LQT1 patients, leads to channel with decreased PIP_2_ sensitivity, suggesting a direct connection between channels-PIP_2_ interactions and the LQT syndrome (Park et al., 2005).

The KCNE1 beta-subunit is critical for a proper activity of KCNQ1 in the heart, and KCNE1 mutations are also associated with a LQT (type 5 LQT syndrome, LQT5). It was shown that neutralization of positive charges located in KCNE1 C-terminus is associated with LQT5 (Lai et al., 2005; Hedley et al., 2009; Kapplinger et al., 2009). A recent study highlighted the importance of PIP_2_ interaction with KCNE1 and suggested that such interaction is critical for a proper function of KCNQ1/KCNE1 in the heart. This study went further by reporting that LQT5 syndrome is directly related to PIP_2_-KCNE1 association, since WT channel complex properties were restored by using higher than normal doses of PIP_2_, thus also confirming the PIP_2_-dependence of LQT5 disease (Li et al., 2011).

Regulation of hERG channels by PIP_2_ has been described in Section “Part 2: Human Ether-a-go-go Related Gene.” PIP_2_ stabilizes hERG open state changing the amplitude and deactivation kinetics (Bian et al., 2001; Rodriguez et al., 2010). In the putative PIP_2_-binding sites, phospholipid anionic heads may interact with intracellular positively charged residues separated by, at least, one aromatic residue (Rosenhouse-Dantsker and Logothetis, 2007; Hernandez et al., 2008). One PIP_2_ interacting site of hERG is localized to the C-terminal part of S6 (residues 883–864; Bian et al., 2004). Interestingly, three type 2 (hERG-related) LQT mutations that lead to substitution or deletion of arginines (at positions 885, 887, and 892) are localized in this area (Napolitano et al., 2005; Tester et al., 2005; Arnestad et al., 2007). It would thus be informative to investigate the activity of these LQT mutant channels to determine a potential PIP_2_ involvement with the LQT2 syndrome.

The importance of PIP_2_ regulation for proper voltage-gated ion channels function deserves thus all our attention. Although no direct connection between the phospholipid and channelopathies has been proven, apart from the LQT studies, the data obtained so far open a wide range of possibilities. An impressive list of phosphoinositide-sensitive channels has been presented in a recent review (Logothetis et al., 2010), and many of them are involved in pathologies (Lehmann-Horn and Jurkat-Rott, 1999). Following the example of hERG above, and knowing that several studies brought further details into the PIP_2_-binding sites on voltage-gated ion channels (Zhang et al., 2003; Oliver et al., 2004; Hernandez et al., 2008; Flynn and Zagotta, 2011; Thomas et al., 2011), we can certainly imagine that other mutations lead to impaired channels-PIP_2_ interaction and thus lead to disease.

## Conflict of Interest Statement

The authors declare that the research was conducted in the absence of any commercial or financial relationships that could be construed as a potential conflict of interest.
